# Evaluating the Impact of Oxidation Heat Treatment and Dual Opaquing Techniques on Enhancing Metal-Ceramic Bond Strength

**DOI:** 10.7759/cureus.68678

**Published:** 2024-09-04

**Authors:** Sandip Rajput, Sneha Raul, Ashwini Dhopte, Chavadapu Kavitha, Pauravi Hegde, Sumit Bhatt

**Affiliations:** 1 Prosthodontics and Implantology, Guru Gobind Singh Dental College and Research Centre, Burhanpur, IND; 2 Prosthodontics, Guru Gobind Singh Dental College and Research Centre, Burhanpur, IND; 3 Oral Medicine and Radiology, Chhattisgarh Dental College and Research Institute, Rajnandgaon, IND; 4 Prosthodontics and Crown Bridge, Mamata Dental College and Hospital, Khammam, IND; 5 Conservative Dentistry and Endodontics, School of Dentistry, DY Patil University, Navi Mumbai, IND; 6 Oral and Maxillofacial Surgery, Rajasthan Dental College and Hospital, Jaipur, IND

**Keywords:** base metal alloy, flexural bond strength, metal-ceramic bond strength, opaque layer, oxidation heat treatment, sem

## Abstract

Background: This research aims to assess the impact of oxidation heat treatment (OHT) and dual opaquing techniques on enhancing the bond strength between metal and ceramic.

Material and method: Eighty rectangular patterns with dimensions of 0.5x3x25 mm (according to ISO 9693-2012) were fabricated in a custom-made silicon mold by using auto-polymerized pattern resin material. These rectangular patterns were cast using base metal alloys. The samples were split into two primary groups: group A, subjected to OHT, and group B, without oxidation treatment. Each primary group was then split up into subgroups according to the application of single layers (group A1, B1) or double layers (group A2, B2) of opaque porcelain. After pre-surface treatment and Ceramco 3 paste opaque application, dentin porcelain (Ceramco 3) was applied to the mid-region of the samples, followed by firing to achieve a standardized thickness. Flexural strength determination was conducted via a three-point bend test performed on the universal testing machine (UTM) (Instron Corp., Model 2519-107, USA), adhering to ISO standard 9693. Post-testing failure types were analyzed by morphological assessment of debonding surfaces via a scanning electron microscope (SEM). The statistical analysis was performed with SPSS version 16, incorporating ANOVA for intergroup analysis and independent t-tests for intragroup comparisons.

Results: Group A2 exhibited the highest mean flexural bond strength (P<0.05) at 41.85 MPa when compared to group A1 at 37.60 MPa, group B2 at 35.47 MPa, and group B1 with the least mean flexural bond strength at 30.41 MPa. SEM observations revealed cohesive bond failure for groups A1, A2, and B2 and adhesive bond failure for groups B1.

Conclusion: It is evident that OHT and opaquing technique are important factors in determining the bond strength of ceramo-metal restorations. When combined, these techniques greatly increase the overall success and durability of metal-ceramic restorations, underscoring their significance in contemporary dental prostheses.

## Introduction

In contemporary dentistry, fixed prosthodontics stands as a cornerstone of restorative dental procedures, offering a range of treatments from single-tooth restorations to full-mouth rehabilitation, often eliciting profound satisfaction for both patients and dentists. This branch of dentistry not only restores function but also transforms unhealthy dentitions into esthetically pleasing occlusions, fostering patient confidence and well-being. The choice of restorative materials for fixed partial prostheses includes options such as all-ceramic, metal-ceramic, and all-metal, each of which meets a particular set of specific clinical needs. While all-metal restorations are strong and durable, they often fall short in terms of esthetics, particularly for anterior restorations. On the other hand, all-ceramic restorations offer natural esthetics but are prone to fracture under high tensile loads. Bridging the gap between these extremes, metal-ceramic prosthesis combines the esthetic appeal of ceramic with the robustness of a metal framework, offering improved fracture resistance and durability [1].

Since their inception in the late 1950s, metal-ceramic prostheses have been widely accepted due to their esthetic appeal, durability, strength, and biocompatibility. Over time, base metal alloys have emerged as a popular option owing to their advantageous mechanical properties and economical considerations compared to precious metals. Notably, the oxidation layer that forms on a base metal alloy is a crucial factor in achieving a strong bond between metal and ceramic.

Oxidation heat treatment (OHT), conducted at temperatures around 960-980°C, serves to remove entrapped gases, eliminate metal surface impurities, and create a crucial oxide layer before ceramic application. The interaction between metal and ceramic is facilitated by this layer, forming the basis of a robust bond [2]. The subsequent application of opaque porcelain further enhances the bond strength by providing a barrier between the metal substructure and the ceramic, optimizing the esthetic outcome while reinforcing the interface. The first opaque layer serves as a wetting layer, while the second layer fills in voids to conceal the metal [3].

Though the metal-ceramic bond is important and ceramo-metal restorations are widely used, the precise mechanisms underlying bond formation remain elusive. Various theories have been proposed, highlighting elements such as van der Waals forces, mechanical interlocking, and chemical bonding [4]. However, achieving a close correlation between the metal and ceramic coefficients of thermal expansion is crucial to reducing stress-induced failures [5].

While traditional approaches have focused on single-layer opaque applications, recent trends have seen the emergence of multilayer techniques, yet the correlation between opaque layering and bond strength remains a subject of debate. Moreover, limited scientific research exists evaluating the bond properties of Ni-Cr alloys subjected to OHT and various opaquing techniques. Therefore, this study endeavors to fill this gap by conducting an in vitro assessment of the impact of the oxidation heat cycle and dual opaquing techniques on the strength of the metal-ceramic bond. Our goal in clarifying the complex interactions among these variables is to offer useful information that can guide clinical procedures and optimize the longevity and performance of metal-ceramic restorations in prosthodontics.

## Materials and methods

**Figure 1:**
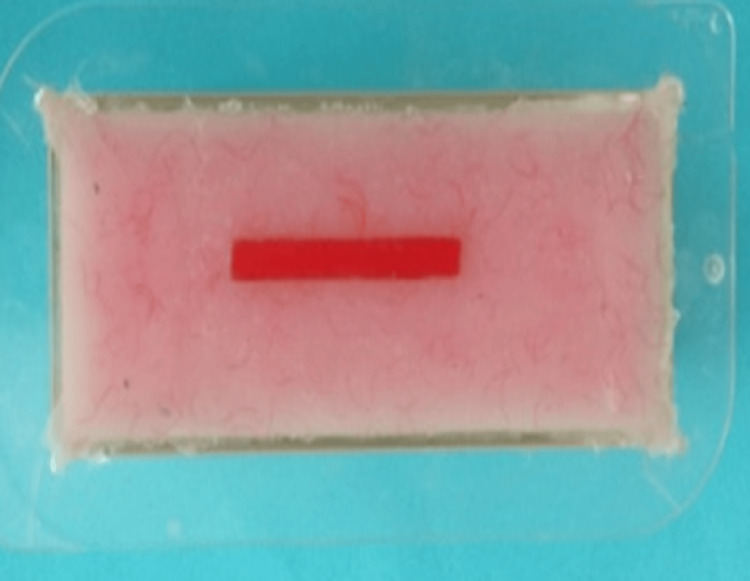
Pattern fabrication using silicon mold and pattern resin

A rectangular tray with a broad flat base loaded with polyvinyl siloxane silicon material (Gingifast, Zhermack, Germany) was used to make an impression of a 0.5 mm thick, 25×3 mm rectangular plastic strip fixed on a glass slab to obtain a silicon mold. A total of 80 rectangular patterns with dimensions of 0.5x3x25 mm were fabricated in a custom-made silicon mold by using pattern resin material (GC; Japan) in a brush-on technique and then flushed with glass slabs (Figure 1).

Castings of these 80 patterns were done using a nickel-chromium base metal alloy (ME Alloy, Dentsply India) following standard procedure. After cleaning and finishing the castings, dimensions were verified for each casting by a digital vernier caliper (Zoom Classic) to ensure specimen standardization. All specimens underwent airborne-particle abrasion with AlO3 (110 µm) at 100 kPa air pressure for 15 seconds, followed by ultrasonic cleaning for five minutes to remove residual investment.

Group A (n=40) specimen underwent OHT, while Group B (n=40) was without OHT. In order to form an oxide layer, the specimens were heated in a ceramic furnace from 500°C to 980°C while under vacuum for three minutes (Table 1).

**Table 1:** Firing protocol and standardized thickness for Ceramco 3 OHT, oxidation heat treatment

Firing cycle	Base temp	Dry time	Temp. raise/min	Final temp	Vacuum set point (in Hg)	Holding time	Standardize thickness
Oxidation heat treatment	500ºC	3 min	100^°^C	980^°^C	29	0	0
Opaque firing (first layer)	500^°^C	3 min	100^º^C	975^°^C	29	0	0.1±0.018 mm
Opaque firing (second layer)	500^º^C	3 min	100^°^C	975^°^C	29	0	0.2±0.031 mm
Dentin firing	650^°^C	5 min	55^º^C	960^º^C	29	0	1.1 mm

Two subgroups were created for each group based on the application of single or double layers of opaque porcelain. The first even layer of opaque paste (Dentsply, Ceramco 3) was applied to the central portion (3×8 mm) of each specimen with a small flat brush, which was then dried for 10 minutes and fired in the furnace following the specifications mentioned in Table 1. The mean thickness of the first opaque layer applied was 0.1±0.018 mm. Then the second layer of opaque paste was applied to the specimens of groups A2 and B2. The second opaque fire followed the same specification mentioned in Table 1. The mean thickness of the second opaque layer applied was 0.2±0.031mm.

**Figure 2:**
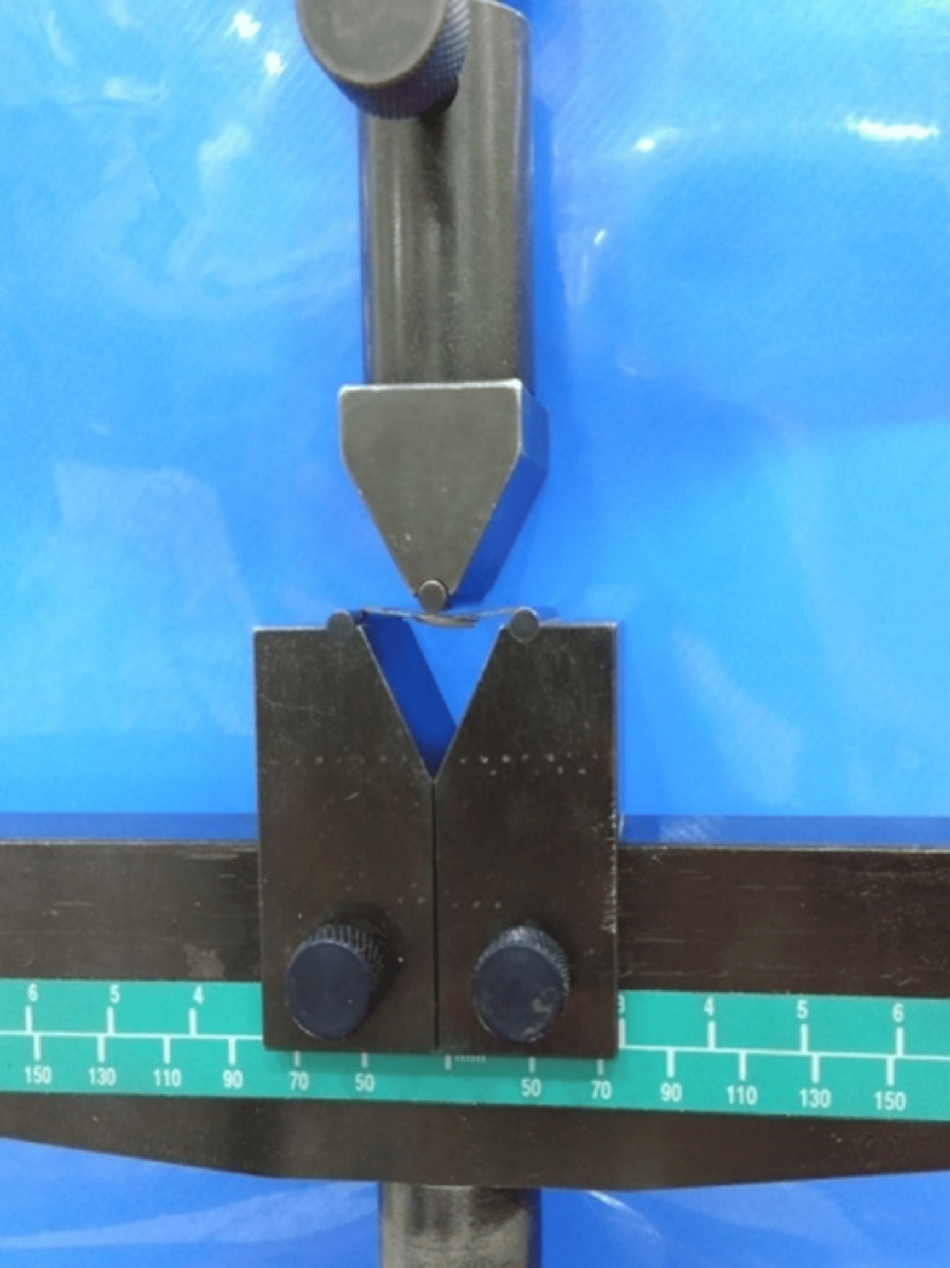
UTM UTM, universal testing machine

 Dentin porcelain (Ceramco 3 Dentsply) was added to the central region of all specimens to obtain a standardized thickness of 1.1 mm using a customized plastic jig, followed by firing in the ceramic furnace. Measurements were made with a vernier caliper at multiple points on the ceramic strip to check the flat, uniform thickness. In accordance with ISO standard 9693, ceramic fracture adherence was assessed via a three-point bend test using a universal testing machine (UTM) (Instron Corp., Model 2519-107, USA). The specimens were positioned symmetrically, and the crosshead speed was set at 1.5 mm/min until the specimens fractured. Failure load was measured in Newtons, and debonding strength was computed using the formula Tb=k×Ffail. A computerized flexural stress versus flexural strain curve was generated, recording the maximum flexural strength in MPa when a sudden drop in the curve was observed at the point of the initial crack (Figure 2).

**Figure 3:**
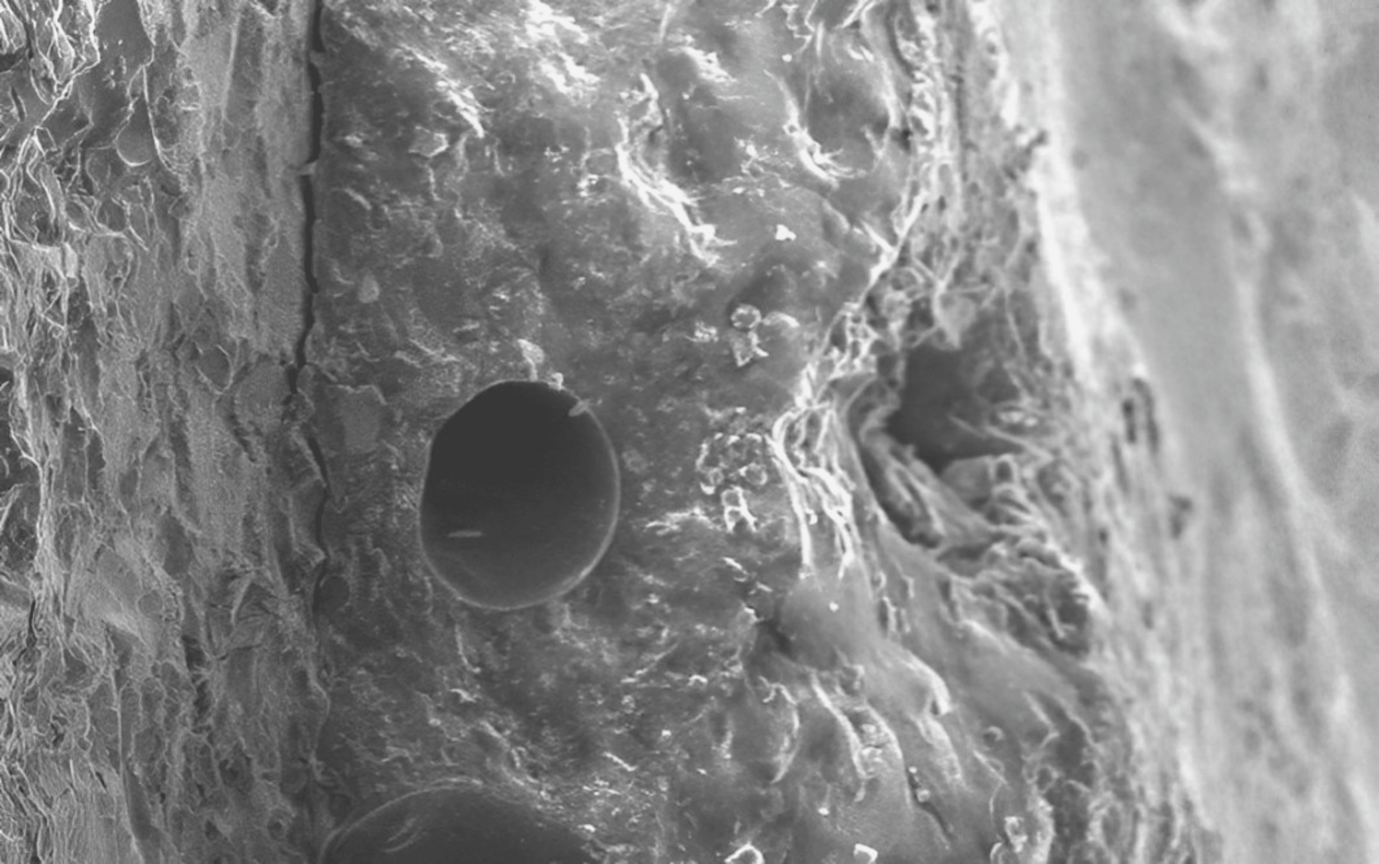
Scanning electron micrographs of A2 group metal-ceramic debonding surface (original magnification ×500)

The morphology of the debonding surfaces of specimens was assessed visually and by scanning electron microscopy (SEM) to analyze failure type. Failure types were divided into three categories: cohesive, adhesive, and mixed-mode. The statistical analysis was performed with SPSS version 16, incorporating ANOVA for intergroup analysis and independent t-tests for intragroup comparisons. The mean and SD were computed to determine statistical significance (Figure 3).

## Results

Group A2 exhibited the highest mean flexural bond strength at 41.85 MPa (SD=7.33), followed by group A1 at 37.60 MPa (SD=7.14), group B2 at 35.47 MPa (SD=7.21), and group B1 with the least mean flexural bond strength at 30.41 MPa (SD=6.13) (Table 2).

**Table 2:** Comparison of flexural bond strength among all the study groups

Groups	N	Mean	SD	p-value
A1: Oxidized single layer	20	37.602000	7.1399901	0.001
A2: Oxidized double layer	20	41.848500	7.3335848	
B1: Non-oxidized single layer	20	30.410000	6.1268512	
B2: Non-oxidized double layer	20	35.467500	7.2120031	

A statistical one-way ANOVA test indicated significant differences (p<0.001) in flexural bond strength among the four groups, leading to the rejection of the null hypothesis and suggesting that flexural bond strength varies with different OHT and opaquing techniques. Subsequent pairwise comparisons using independent t-tests revealed that while group A2 demonstrated higher mean flexural bond strength compared to group A1, the difference was not statistically significant (p<0.71), indicating no variation in bond strength with the two opaquing techniques of the oxidized groups (Table 3).

**Table 3:** Comparison of flexural bond strength among oxidized single layer versus oxidized double layer

Groups	N	Mean	SD	Difference	p-value
A1: Oxidized single layer	20	37.602000	7.1399901	-4.2465	0.071
A2: Oxidized double layer	20	41.848500	7.3335848		

However, significant differences were observed in flexural bond strength between group B2 and group B1 (p<0.022) (Table 4).

**Table 4:** Comparison of flexural bond strength among non-oxidized single layer versus non-oxidized double layer

Groups	N	Mean	SD	Difference	p-value
B1: Non-oxidized single layer	20	30.410000	6.1268512	-5.0575	0.022
B2: Non-oxidized double layer	20	35.467500	7.2120031		

Additionally, group A1 showed significantly higher flexural bond strength compared to group B1 (p<0.002) (Table 5), and group A2 exhibited significantly higher bond strength than group B2 (p<0.009) (Table 6).

**Table 5:** Comparison of flexural bond strength among oxidized single layer versus non-oxidized single layer

Groups	N	Mean	SD	Difference	p-value
A1: Oxidized single layer	20	37.602000	7.1399901	7.1920	0.002
B1: Non-oxidized single layer	20	30.410000	6.1268512		

**Table 6:** Comparison of flexural bond strength among oxidized double layer versus non-oxidized double layer

Groups	N	Mean	SD	Difference	p-value
A2: Oxidized double layer	20	41.848500	7.3335848	6.3810	0.009
B2: Nonoxidized double layer	20	35.467500	7.2120031		

Furthermore, all specimens met the ISO 9693:2012 (E) requirements for bond strength greater than 25 MPa [2,4,6,7]. SEM observations revealed distinct characteristics of bond failure among the different groups. In groups A1, A2, and B2, cohesive bond failure was observed, suggesting that the bond between the ceramic and metal was stronger than the bond within the ceramic itself. Sufficient porcelain remained on the metal surface, suggesting robust bonding between the oxidized Ni-Cr alloy and the ceramic. This observation aligns with the higher bond strength values recorded for these groups. Furthermore, the presence of more porcelain on the oxidized fracture surfaces further supported the notion of enhanced bonding between the oxidized Ni-Cr alloy and the porcelain, contributing to the higher bond strength values observed. In contrast, group B1 exhibited adhesive modes of failure between the alloy and the opaque layer. This finding suggests minimal bond strength, suggesting that the bond between the ceramic and the metal was weaker compared to cohesive bonding within the ceramic.

## Discussion

The mean bond strength of the four groups varied significantly, indicating that the opaquing technique and the OHT are important determinants of the ceramo-metal restoration's bond strength. In this investigation, oxidized groups (groups A1, A2) showed stronger bonds than non-oxidized groups (groups B1, B2). This could be because the binding strength is determined by the thickness and presence of an oxide layer. An oxide layer that is either abnormally thick or nonexistent is the cause of the weak bond strength. Two reasons for the increased bond strength in oxidized groups were van der Waals forces, chemical bonding, compressive forces, and mechanical bonding [1,8-10]. The crucial element, chemical bonding, describes how metal oxide and porcelain interact chemically [2]. With no discernible differences between them (p=0.71), the mean flexural bond strength of the double-layer opaque (A2) in the oxidized group was higher than that of the single-layer opaque (A1), indicating that both opaquing techniques perform similarly in terms of flexural bond strength under oxidized conditions. These outcomes concur with those of DG Jochen and Philip C. Rake [3,11]. Significant variations in bond strength were found by pairwise comparisons between oxidized single-layer (A1) and non-oxidized single-layer (B1) groups, as well as between non-oxidized double-layer (B2) and non-oxidized single-layer (B1) groups. The non-oxidized single-layer group (B1) had the lowest bond strength, which was partly explained by the oxide layer's absence [12-15]. Furthermore, notable distinctions were seen between the groups with oxidized double-layers (A2) and those without (B2). The non-oxidized double-layer group exhibited a significantly stronger bond strength than the non-oxidized single-layer group. This could be explained by the use of paste opaque as the opaque layer application method in this investigation. Paste opaque is a premixed "oily" material that requires more time during the pre-heating phase to evaporate the "oily" pigment (±8 minutes) [13]. Oxidation of the alloy occurred before the porcelain began to flow, during the beginning of the firing cycle [3]. Specimens in group B2 were not oxidized, as in this study, although they did undergo an additional fire cycle during the second opaque firing cycle. The present study's results are consistent with those of Philip et al., who examined the effects of two oxidizing techniques and two metal surface conditions on the metal-ceramic bond strengths of base metal alloys, silver-free gold-palladium alloys, and gold platinum palladium alloys [11]. Rake et al. found that the oxidized surface group significantly increased the bond strengths of these alloys [11]. Standardized plates measuring 0.5x3x25 mm and manufactured in accordance with ISO standard 9693 were used in this study [16]. Due to the benefit of eliminating faults that could be introduced during the duplicating phase, a bespoke mold was created using the additional silicone for the creation of patterns, hence reducing the variation within each group [17,18]. The patterns were created using pattern resin, which has better dimensional stability and less casting shrinkage than casting waxes [19]. A Ni-Cr alloy was selected for this study in order to serve as a representative example of a typical metal-ceramic alloy due to its more advantageous mechanical properties, affordability, and ease of casting. The purpose of OHT, which is carried out at temperatures between 960°C and 980°C, is to release trapped gases, remove surface contaminants from metal, and produce an essential oxide layer [20]. The oxide that was on the metal's surface helped the porcelain stick to it [21]. The binding strength was 30% less if the oxide coating on the alloy surface was removed [22]. The primary purposes of the opaque layer are to create a sufficiently reflecting and absorbent layer to hide the metal substructure and to effectively attach the metal to the ceramic. For opaque materials that are easily found on the market, there are several application techniques offered, including paste, spray, and powder-liquid. Using the paste opaque technique is easier and results in a consistent coating on sharp surfaces like the metal substructure incisal edges. While the second layer hides any imperfections to mask the metal, the first layer serves as a wetting layer [3]. The coefficient of thermal expansion (CTE) for the base metal alloy (MEalloy) utilized in the study is 14.1×10⁻⁶ K⁻¹, but the CTE for Ceramco 3, which was employed with the alloys, varies between 13.9 and 14.9×10⁻⁶ K⁻¹. The metal's CTE should be slightly higher (0.5×10^6^ K ¯ 1) than the ceramic since the ceramic is meant to compress the interface and form a strong connection. Using a specially made plastic jig, dentin (body) porcelain application was created to mimic the standard ceramic thickness for dental crowns. The metal-ceramic bond strength has been measured using a variety of methods, such as the traction test, shear bond strength, three-point flexural bond strength, and four-point flexural bond strength. Numerous experiments, such as the traction test, shear bond strength, four-point flexural bond strength, and three-point flexural bond strength, have been used to evaluate the metal-ceramic bond strength [4,14,23]. Despite difficulties in accurately determining the true bond strength, the three-point flexural test is one of the most used among them. The reason this test is preferred is that it closely resembles clinical scenarios by simultaneously subjecting the specimens to traction, compression, and shear bond strength [24]. Furthermore, the International Organization for Standardization recommends using this three-point test to determine the ceramo-metal bond [16]. SEMs were used in this study to further evaluate the strength of the metal-ceramic bond. They showed that the B1 group had adhesive fracture modes, whereas the A1, A2, and B2 groups had cohesive modes. While the adhesive mode of failure between the oxide layer and the alloy was seen, the cohesive mode of failure suggests that there is still enough porcelain on the metal surface. The optimal situation was cohesive fracture mode, which indicated that more forces were needed to separate the ceramic and metal because the bond between them was stronger than that within the ceramic [2,8]. The current study's results, based on the experimental settings, demonstrated that all samples exceeded 25 MPa, satisfying the minimum bond strength standards stipulated by ISO 9693 and ADA Specification 38 [6,7]. The forces used on the specimens in the current laboratory research did not precisely replicate clinical settings, which was a drawback. Consequently, additional in vivo clinical trials are needed to determine how well this approach variation performs intraorally.

## Conclusions

In conclusion, the data from this study support the assertion that OHT and the opaquing technique are key factors in determining the metal-ceramic bond strength. These techniques not only enhance bonding between the ceramic and metal but also optimize the interface between the two materials, thereby ensuring durable and long-lasting restorations. Clinically, the insights gained from this study can guide the selection of the most effective surface treatment protocols for achieving optimal bond strength in metal-ceramic restorations, ultimately leading to improved treatment outcomes for patients.
